# Monocyte count combined with GTVnx is an independent prognostic factor in non-metastatic nasopharyngeal carcinoma receiving radiotherapy

**DOI:** 10.3389/fonc.2025.1541212

**Published:** 2025-04-08

**Authors:** Li-Na Yang, Yun-Rui Song, Huan Zhang, Hong-Lei Tu, Ming-Yue Lu, De-Qing Liu, Jiang-Dong Sui, Dan Li, Yue Xie, Ying Wang

**Affiliations:** Radiation Oncology Center, Chongqing University Cancer Hospital, School of Medicine, Chongqing University, Chongqing, China

**Keywords:** peripheral blood monocyte count, GTVnx, nasopharyngeal carcinoma, radiotherapy, overall survival

## Abstract

**Background and purpose:**

The relationship between peripheral blood monocyte count and primary gross tumor volume with survival prognosis in newly diagnosed nasopharyngeal carcinoma(NPC) patients who received radiotherapy remains unclear. Therefore, We conducted a cohort study to assess the association of peripheral blood monocyte count and primary gross tumor volume with survival outcomes in newly diagnosed non-metastatic NPC patients who received radiotherapy.

**Materials/methods:**

We included newly diagnosed non-metastatic NPC patients who underwent radiotherapy in our hospital from January 2013 to December 2015. General clinical characteristics such as age, gender, ECOG score and tumor stage, peripheral blood monocyte count, lymphocyte count, white blood cell count (WBC), neutrophil count, radiotherapy technology, total radiotherapy days, gross tumor volume of nasopharyngeal carcinoma (GTVnx) and gross tumor volume of cervix node (GTVnd) of patients before radiotherapy, and whether chemotherapy was induced were recorded. The primary endpoint was overall survival, the secondary endpoint was progression-free survival. Univariate and multivariate COX regression were used to analyze the relationship among peripheral blood monocyte count, GTVnx, and survival outcomes. Spearman correlation analysis was used to analyze the correlation between risk factors. Based on the independent risk factors for OS, we further divide patients into three different risk groups, and the differences in clinical and therapeutic indicators and survival outcomes between the three groups were analyzed using a one-way analysis of variance.

**Results:**

A total of 448 participants were included in the study, the median follow-up time was 74.3 months. Of these, 97 (21.7%) died. In the univariate and multivariate Cox regression analyses, peripheral blood monocyte count and GTVnx were independently associated with OS. The high monocyte count and GTVnx were associated with the poor OS and PFS. Correlation analysis showed that monocyte count was positively correlated with WBC, platelet, and neutrophil. GTVnx was positively correlated with platelet, neutrophil, and Epstein-Barr virus before treatment. Survival curves significantly differed among patients in different risk groups for OS (p = 0.0008) and PFS (p = 0.0007). Besides, For every increase in monocyte unit count, the OS and PFS risks of patients in the low GTVnx group increased by 2.64 and 2.31 folds, respectively.

**Conclusions:**

Peripheral blood monocyte count combined with GTVnx is an independent predictor for overall survival and progression free-survival in newly diagnosed non-metastatic NPC patients who received radiotherapy. The benefit of patients with GTVnx< 28.5cm^3^ could be remarkably attenuated by the high monocyte count.

## Introduction

Nasopharyngeal carcinoma (NPC) is one of the head and neck malignancies, which arises in the upper lining epithelium of the nasopharynx and is often observed at the pharyngeal recess (1). According to the International Agency for Research on Cancer, in 2020, there were about 133,354 new cases and 80,008 new deaths of nasopharyngeal carcinoma, accounting for only 0.7% of all cancers diagnosed and 0.8% of all cancers deaths (2). Although the incidence and mortality of nasopharyngeal carcinoma are not high compared with other cancers, it has a remarkable geographical distribution, >70% of new cases are in East and Southeast Asia. It is well known that radiotherapy is the standard treatment for this malignancy, because of the high sensitivity to ionizing radiation and anatomical location (1). Combined with the current standard chemoradiotherapy regimens, the survival of nasopharyngeal carcinoma has been greatly improved, according to reports, the estimated 5-year overall survival (OS) after radiotherapy for stage I-II NPC is 98% and 92%, local failure-free survival (FFS) is 98% and 94%, and distant FFS is 98% and 91% (3).

Unfortunately, although the use of concurrent chemo-radiotherapy (CCRT) as the standard treatment for locally advanced nasopharyngeal carcinoma has significantly improved survival, the results for locally advanced disease are not satisfactory (4). Therefore, an efficient, inexpensive, and valuable biochemical index is needed to predict the prognosis of patients with NPC and to promote individualized treatment to provide a better prognosis for patients. Inflammatory response plays an important role in the occurrence and development of cancer, and it has even been reported that inflammatory cells have better prognostic importance for tumors compared with histopathological results (5). In previous studies, many inflammatory markers such as lymphocytes, neutrophils, neutrophil to lymphocyte ratio (NLR), and lymphocyte to monocyte ratio (LMR) are associated with cancer prognosis (6, 7).

Monocytes, which can differentiate into macrophages and myeloid dendritic cells, are key immune cells in the inflammatory response (8). The roles of monocytes are complicated, they act as a bridge between innate and adaptive immune responses, influencing the tumor microenvironment by inducing increased immune tolerance, angiogenesis, and tumor cell dissemination through multiple mechanisms (9). It has been reported to be independently associated with the prognosis of various tumors, such as non-small cell lung cancer, squamous cell carcinoma of the head and neck, prostate cancer, and metastatic nasopharyngeal carcinoma (10–13).

Gross tumor volume(GTV) is an intuitive quantitative reflection of tumor burden, the longer the malignancy occurs, the larger the GTV, the wider the anatomical range of possible invasion, and the worse the therapeutic effect. Several studies have shown that GTV is an important prognostic indicator of malignant tumors (14, 15). GTV included gross tumor volume of nasopharyngeal carcinoma (GTVnx) and gross tumor volume of cervix node(GTVnd). Sze WM et al. (16) confirmed that every 1 cm^3^ increase in nasopharyngeal carcinoma volume caused a 1% increased risk of local recurrence.

The purpose of the study is to determine the prognostic significance of pre-treatment peripheral blood monocyte count and GTVnx in newly diagnosed non-metastatic NPC patients treated with radiotherapy.

## Materials and methods

### Study design and participants

We included 448 newly diagnosed non-metastatic nasopharyngeal carcinoma(NPC) patients who received radiotherapy at Radiation Oncology Center, Chongqing University Cancer Hospital from January 2013 to December 2015 in this study.

Inclusion criteria: (1) Patients who are first diagnosed as NPC by pathology and without distant metastasis. (2) Age Over 18 and under 80. (3) ECOG Score ≤ 2. (4) Routine blood test was performed one week before radiotherapy. (5) No complications that seriously affect peripheral blood cells (i.e., acute or chronic infection, hematological system diseases, any immune deficiency disease, and diseases requiring treatment with glucocorticoid replacement). (6) The radiotherapy plan was clearly filled out, the total duration of radiotherapy did not exceed 70 days, and the total radiotherapy dose in the nasopharyngeal region was≥ 66 Gy.

Informed consent was obtained from all participants at baseline and all procedures were performed in accordance with our local guidelines and clinical regulations. The study was conducted in accordance with the Declaration of Helsinki, and the protocol was approved by the Ethics Committee of The Chongqing University Cancer Hospital (No. CZLLYJ0252).

### Data collection and laboratory measurements

Medical and social history were collected and recorded. Include age, gender, smoking, drinking, pre-treatment Epstein-Barr virus DNA, Charlson Comorbidity Index(CCI), Eastern Cooperative Oncology Group (ECOG) score, TNM stage (based on the American Joint Committee on Cancer (AJCC) 7th edition), induction chemotherapy (no chemotherapy was performed within 3 weeks before the peripheral blood cells counts were recorded), radiotherapy parameters (total duration of radiotherapy, GTVnx and GTVnd (cm^3^), body Dmean(Gy), bone Dmean(Gy), and radiation technology). After fasting for at least 8 hours overnight, all patients underwent fasting vein blood collection the next morning and were sent to the laboratory within one hour after blood collection. The blood routine was tested by an automatic hematology analyzer. Baseline peripheral blood cell counts including peripheral blood monocyte count, lymphocyte count (LC), white blood cell count (WBC), hemoglobin (Hgb), and neutrophil count were measured and recorded. LMR and NLR are defined as the ratio of lymphocyte count to monocyte count and neutrophil count to lymphocyte count. Post-treatment peripheral blood monocyte count were also be recorded after one month, two months and three months of the radiotherapy.

### Outcome and follow-up

The primary endpoint of this study was the overall survival (OS), which was defined as the time from the first day of radiotherapy to all-cause death or the last follow-up visit. The secondary endpoint was progression-free survival (PFS), which calculated the time from the start of treatment to the first failure in any part or death for any reason or final follow-up. Patients were examined every 3 months for the first 2 years after completing treatment, every 6 months for the third to fifth years, and annually after 5 years. Survival information were collected from patients’ hospital records and periodic telephone follow-up records.

### Statistical analysis

In this study, SPSS 22.0 statistical software was used for data analysis. The measurement data were represented by mean ± standard deviation (SD), and the counting data were represented by number and percentage. An Independent sample t-test or Pearson Chi-square test was used for the comparison between the two groups. Comparison among three groups using one-way analysis of variance. Then Kaplan-Meier method was used to draw the survival curves. Univariate Cox regression analyses were used to evaluate the correlation between parameters and OS. Multivariate Cox regression analyses were used for the evaluation of OS and monocyte count, GTVnx. Spearman correlation analysis was used to analyze the correlation between risk factors. Two-tailed p values <0.05 were considered statistically significant.

## Results

A total of 448 NPC patients were included in the final analysis. Among them, 319 (71.2%) were males, 416 (92.9%) patients underwent intensity-modulated radiation therapy (IMRT) and 32 (7.1%) patients underwent three-dimensional conformal radiation therapy (3D-CRT). There were 428 (95.5%) patients with ECOG score 0-1 and 20 (4.5%) patients with ECOG score 2. The mean age of all NPC patients was 51 years, 425 of whom underwent induction chemotherapy before, and 110 (24.6%), 233 (52%), and 105 (23.4%) patients with comprehensive stage II, III, and IV, respectively. The median follow-up time was 74.3 months (interquartile range 24.7-87.8). At the last follow-up, 97 people (21.7%) had died from all causes. Radiotherapy parameters for all patients were as follows: mean total radiotherapy days were 47 days, mean total radiotherapy dose was 71Gy, mean body Dmean (Gy) radiotherapy dose was 19.5Gy, mean GTVnx was 37.6cc, GTVnd was 28.6cc, and mean bone dose was 21.3Gy. Baseline routine blood parameters were as follows: mean blood white blood cell count of 5.71, mean monocyte count of 0.31, mean lymphocyte count of 1.51, mean lymphocyte count to monocyte count ratio of 13.5, mean Hemoglobin is 135, mean neutrophil count is 3.77 (**Table 1**).

**Table 1:** Clinical characteristics of the total nasopharyngeal carcinoma patients.

Characteristics	Values
Age, years	51.19 ± 10.15
Gender	
Male Female	319(71.2) 129(28.8)
Radiotherapy technique	
IMRT	416(92.9)
3D-CRT	32(7.1)
ECOG	
0	36(8.0)
1	392(87.5)
2	20(4.5)
CCI	
2 3 4 ≥5	170(37.9) 151(33.7) 86(19.2) 41(9.2)
Induction chemotherapy	425(94.9)
Concurrent chemoradiotherapy	9(2.0)
T-stage	
1	40(8.9)
2	208(46.4)
3	127(28.3)
4	73(16.3)
N-stage	
0	36(8.0)
1	183(40.8)
2	190(42.4)
3	39(8.7)
Clinical stage	
II	110(24.6)
III	233(52.0)
IVa Pre-treatment Epstein-Barr virus DNA Positive Negative Unknown	105(23.4) 65(14.5) 110(24.6) 273(60.9)
Total radiotherapy days	46.70 ± 5.75
Total radiotherapy dose(Gy)	70.96 ± 1.49
Body Dmean(Gy)	19.49 ± 3.34
GTVnx(cm^3^)	37.60 ± 29.62
GTVnd(cm^3^)	28.63 ± 38.87
Bone Dmean(Gy)	21.30 ± 15.49
OS time(m) PFS time(m)	59.46 ± 33.83 54.95 ± 35.81
WBC(×10^9^/L)	5.71 ± 2.17
Monocyte(×10^9^/L)	0.31 ± 0.25
Lymphocyte(×10^9^/L)	1.51 ± 0.54
LMR(Lymphocyte to monocyte ratio) NLR(Neutrophil to Lymphocyte ratio)	13.49 ± 23.01 2.87 ± 2.28
Hemoglobin(g/L)	135.13 ± 16.78
Neutrophil(×10^9^/L)	3.77 ± 1.92
Smoking	199(44.4)
Drinking	115(25.7)

Values are mean ± SD, n(%).

In the univariate Cox regression analysis (**Table 2**) gender, radiotherapy technique, induction chemotherapy, concurrent chemoradiotherapy, EBV DNA, ECOG, CCI, smoking, and drinking are categorical variable), parameters such as monocyte count [HR 2.30, 95%CI (1.13-4.69)], age[1.03(1.01-1.05)], GTVnx[1.01(1.00-1.01)], CCI[1.75(1.17-2.63)], NLR[1.07(1.01-1.13)], Hgb[0.99(0.97-0.99)] were shown to be significantly associated with OS. In the multivariate Cox regression analyses (**Table 2**), monocyte count[2.13(1.02-4.44)] and GTVnx[1.01(1.00-1.02)] were independently associated with OS.

**Table 2:** Univariate and multivariable Cox regression analysis.

Variables	Univariate analysis		Multivariable analysis	
	Hazard ratio (95% CI)	P	Hazard ratio (95% CI)	P
Monocyte (×10^9^/L)	2.30 (1.13-4.69)	0.022	2.13 (1.02-4.44)	0.044
Age	1.03 (1.01-1.05)	0.008	1.02 (0.99-1.05)	0.177
Gender Radiotherapy technique Induction chemotherapy Concurrent chemoradiotherapy Pre-treatment Epstein-Barr Virus DNA	0.83 (0.53-1.31) 1.20 (0.49-2.97) 1.12 (0.49-2.57) 0.41 (0.06-2.94) 1.65 (0.94-2.90)	0.417 0.686 0.784 0.299 0.081		
Total radiotherapy days Total radiotherapy dose (Gy)	1.02 (0.99-1.06) 1.11 (0.96-1.27)	0.211 0.161		
BodyDmean (Gy)	1.05 (0.99-1.12)	0.173		
GTVnx (cm^3^)	1.01 (1.00-1.01)	0.021	1.01 (1.00-1.02)	0.030
Bone Dmean (Gy)	1.00 (0.99-1.01)	0.380		
ECOG CCI	0.84 (0.48-1.48) 1.75 (1.17-2.63)	0.554 0.007	1.25 (0.69-2.27)	0.462
Smoking	1.34 (0.90-2.00)	0.149		
Drinking	1.41 (0.92-2.16)	0.116		
Lymphocyte (×10^9^/L)	0.99 (0.98-1.01)	0.220		
NLR	1.07 (1.01-1.13)	0.043	1.06 (0.99-1.12)	0.077
Hemoglobin (g/L)	0.99 (0.97-0.99)	0.017	0.99 (0.98-1.00)	0.229

**Figure 1:**
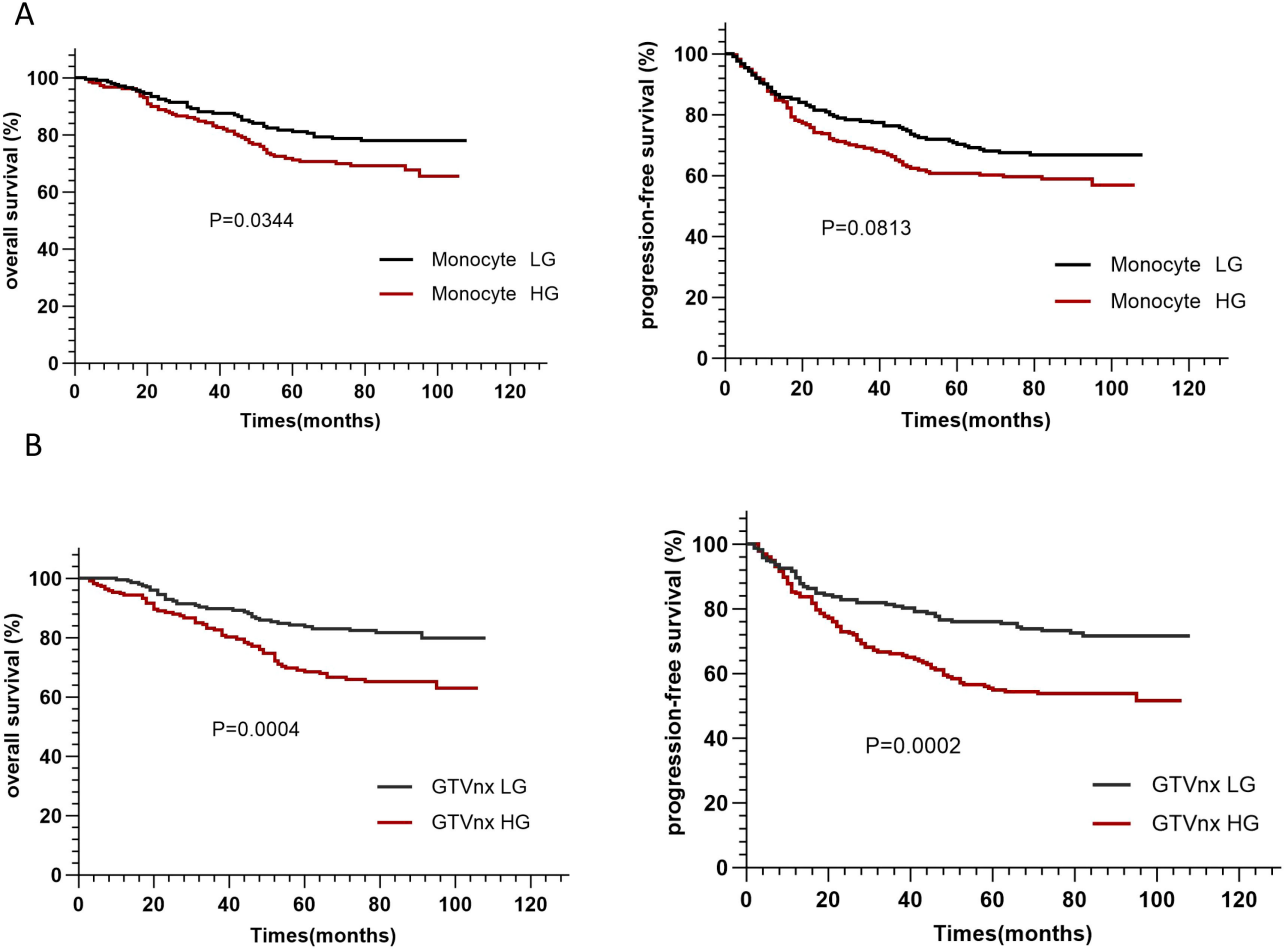
The Kaplan–Meier survival curves of monocyte count **(A)**, GTVnx **(B)** based on the overall survival (OS) and progression-free survival (PFS) in NPC patients. (Monocyte LG: monocyte count <0.29×10^9^/L, Monocyte HG: monocyte count ≥0.29×10^9^/L, GTVnx LG: GTVnx <28.5cm^3^, GTVnx HG: GTVnx ≥28.5cm^3^.).

To further confirm the predictive effect of monocyte count and GTVnx on OS and PFS, we divided patients into low and high groups based on baseline median monocyte count and GTVnx. As shown in **Figure 1**, the high monocyte count group and high GTVnx group were associated with poor OS and PFS. In the two-independent sample T-test, there were no statistical differences in other clinical parameters between the two groups except baseline monocyte count, GTVnx, WBC, LMR, and NLR (**Table 3**). The clinical characteristics between the high monocyte count group and low monocyte count group were compared, and there were no statistical differences in age, sex, pre-treatment Epstein-Barr virus DNA, ECOG score, tumor stage, smoking history, drinking history, CCI, radiotherapy method, total radiotherapy days, whether chemotherapy was induced, radiotherapy and hematology indexes between the two groups.

**Table 3:** Comparison of clinical characteristics between two monocyte count groups.

	Monocyte-low (223)	Monocyte-high (225)	P value
Age, years	50.85 ± 10.21	51.53 ± 10.11	0.959
Gender			0.644
Male Female	161 (72.2) 62 (27.8)	158 (70.2) 67 (29.8)	
ECOG			0.670
0	18 (8.1)	18 (8.0)	
1	197 (88.3)	195 (86.7)	
2 CCI 2 3 4 ≥5	8 (3.6) 73 48 11 91	12 (5.3) 83 77 38 27	0.716
Induction chemotherapy	216 (96.9)	209 (92.9)	0.057
T-stage			0.500
1	23 (10.3)	17 (7.6)	
2	105 (47.1)	103 (45.8)	
3	57 (25.6)	70 (31.1)	
4	38 (17.0)	35 (15.6)	
N-stage			0.336
0	13 (5.8)	23 (10.2)	
1	93 (41.7)	90 (40.0)	
2	99 (44.4)	91 (40.4)	
3	18 (8.1)	21 (9.3)	
Clinical stage			0.752
2	57 (25.6)	53 (23.5)	
3	112 (50.2)	121 (53.8)	
4	54 (24.2)	51 (22.7)	0.408
Pre-treatment Epstein-Barr virus DNA (Positive)	30 (6.7)	35 (7.8)	
Total radiotherapy days	46.52 ± 5.81	46.87 ± 5.71	0.766
Total radiotherapy dose (Gy)	70.96 ± 1.51	70.95 ± 1.47	0.943
Body Dmean (Gy)	20.00 ± 3.19	18.99 ± 3.42	0.501
GTVnx (cm^3^)	35.78 ± 26.24	39.39 ± 32.58	0.005
GTVnd (cm^3^)	28.79 ± 39.00	28.48 ± 38.83	0.692
Bone Dmean (Gy)	21.92 ± 15.41	20.68 ± 15.58	0.843
WBC (×10^9^/L)	5.23 ± 1.92	6.19 ± 2.30	0.005
Monocyte (×10^9^/L)	0.13 ± 0.09	0.49 ± 0.22	0.000
Lymphocyte (×10^9^/L)	1.39 ± 0.50	1.62 ± 0.55	0.233
LMR	23.42 ± 29.49	3.69 ± 1.47	0.000
NLR	3.07 ± 2.71	2.66 ± 1.73	0.000
Hemoglobin (g/L)	135.35 ± 16.05	134.91 ± 17.50	0.254
Neutrophil (×10^9^/L)	3.60 ± 1.88	3.93 ± 1.94	0.424
Smoking	94 (42.5)	106 (47.1)	0.335
Drinking	57 (25.7)	58 (25.8)	0.936

Values are mean ± SD, n (%).

**Figure 2:**
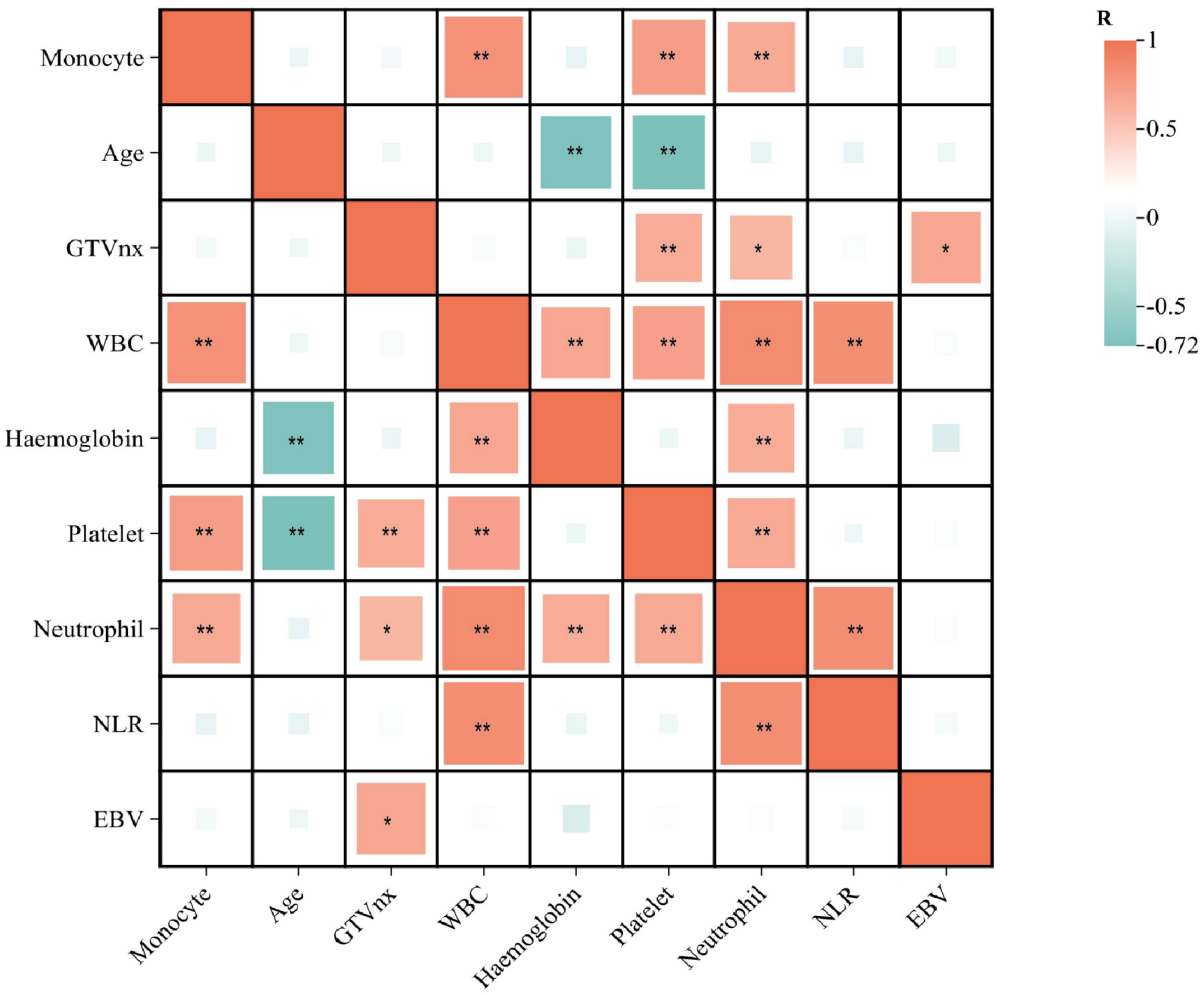
Risk factor correlation matrix diagram (*=P<0.05; **=P<0.01). This figure was drawn using ChiPlot (https://www.chiplot.online/) (accessed on March 2025).

The correlation analysis of **Figure 2** showed that peripheral blood monocyte count was positively correlated with WBC, platelet, and neutrophil (P<0.05). GTVnx was positively correlated with platelet, neutrophil, and Epstein-Barr virus before treatment (P<0.05). As shown in **Supplementary Figure S1**, the higher the peripheral blood monocyte count at 1 month, 2 months, and 3 months after radiotherapy, the worse the OS and PFS, which supports our previous conclusion.

**Figure 3:**
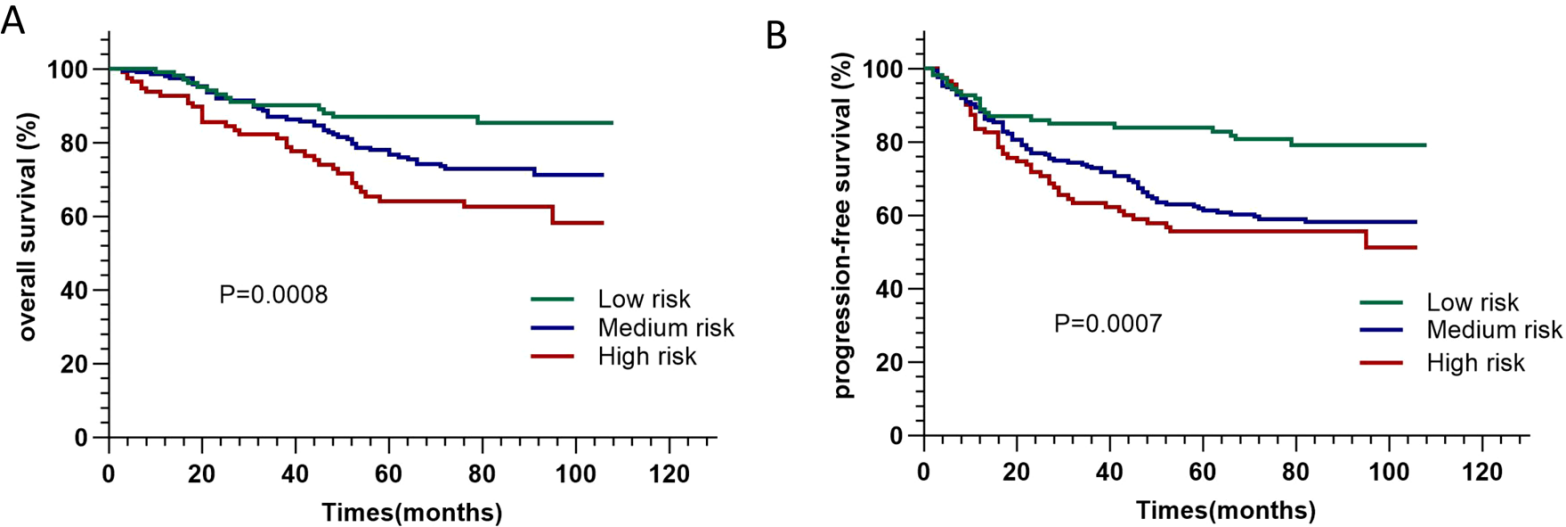
Kaplan-Meier curves of overall survival **(A)** and progression-free survival **(B)** of patients in different risk groups.

Based on the independent risk factors (monocyte count ≥ 0.29×10^9^/L (median), GTVnx ≥ 28.5cm^3^ (median)) for OS, we further divide patients into three different risk groups: low-risk group (with <1 risk factor), medium-risk group (with 1 risk factors), and high-risk group (with 2 risk factors). Survival curves were significantly different among patients in different risk groups for OS (p = 0.0008) and PFS (p = 0.0007) (**Figure 3**). The characteristics of patients in the different risk groups are shown in **Table 4**. As shown in **Table 5**, patients in the GTVnx low group had a 2.64-fold and 2.31-fold increased risk of OS and PFS for every increase in the unit count of monocytes, and in the GTVnx high group had a 1.94-fold and 1.09-fold increase, respectively. In the low monocyte count group, the risk of OS and PFS increased by 1.01 times per unit volume increase of GTVnx, and in the high monocyte count group, GTVnx increases the risk of OS and PFS by 1.00 times per unit volume increase (**Supplementary Table 1**). Altogether, the prognostic benefit of patients with GTVnx< 28.5cm^3^ could be remarkably attenuated by the high monocyte count.

**Table 4:** Characteristics of patients in different risk groups.

	Low-risk group (117)	Medium-risk group(215)	High-risk group (116)	P value
Age, years	51.02 ± 9.59	50.96 ± 10.51	51.66 ± 10.02	0.827
Male	78(67.2)	155(70.9)	86(74.1)	0.178
ECOG				0.052
0	13(11.2)	17(7.9)	6(5.2)	
1	99(85.3)	189(87.9)	103(88.8)	
2	4(3.5)	9(4.2)	7(6.0)	
CCI				0.937
2 3 4 ≥5	39(33.3) 42(35.9) 21(17.9) 15(12.8)	89(41.4) 64(29.8) 45(20.9) 17(7.9)	42(36.2) 45(38.8) 19(16.4) 10(8.6)	
Induction chemotherapy	111(95.6)	203(94.4)	111(95.6)	0.662
T-stage				0.000
1	18(15.5)	20(9.3)	1(0.8)	
2	75(64.7)	103(47.9)	30(25.9)	
3	14(12.1)	58(26.9)	56(48.3)	
4	9(7.8)	34(15.8)	29(25.0)	
N-stage				0.610
0	7(6.0)	19(8.8)	10(8.6)	
1	55(47.4)	85(39.5)	43(37.1)	
2	47(40.5)	92(42.8)	51(44.0)	
3	7(6.0)	19(8.8)	12(10.3)	
Clinical stage				0.000
2	48(41.4)	51(23.7)	11(9.5)	
3	51(44.0)	116(54.0)	66(56.9)	
4	17(14.6)	48(22.3)	39(33.6)	0.151
Pre-treatment Epstein-Barr virus DNA(Positive)	12(10.3)	24(11.2)	29(25)	
Total radiotherapy days	45.59 ± 5.20	46.90 ± 5.82	47.48 ± 6.03	0.034
Total radiotherapy dose (Gy)	70.74 ± 1.75	71.03 ± 1.29	71.04 ± 1.55	0.189
Body Dmean (Gy)	19.19 ± 2.95	19.78 ± 3.35	19.25 ± 3.66	0.203
GTVnx(cm^3^)	17.15 ± 7.16	36.19 ± 26.44	60.65 ± 33.01	0.000
GTVnd(cm^3^)	22.90 ± 26.31	30.34 ± 40.40	31.51 ± 46.17	0.201
Bone Dmean (Gy)	19.96 ± 3.54	21.78 ± 15.68	21.72 ± 21.41	0.564
WBC(×10^9^/L)	5.40 ± 1.92	5.52 ± 2.15	6.41 ± 2.31	0.000
Monocyte(×10^9^/L)	0.16 ± 0.13	0.29 ± 0.23	0.49 ± 0.25	0.000
Lymphocyte(×10^9^/L)	1.42 ± 0.48	1.49 ± 0.54	1.61 ± 0.57	0.019
LMR	22.36 ± 30.79	13.96 ± 22.37	3.80 ± 1.97	0.000
NLR	3.01 ± 2.59	2.86 ± 2.43	2.77 ± 1.56	0.712
Hemoglobin(g/L)	136.42 ± 15.13	135.08 ± 16.65	134.02 ± 18.58	0.550
Neutrophil(×10^9^/L)	3.65 ± 1.97	3.66 ± 1.88	4.10 ± 1.91	0.102
Smoking	38(32.8)	99(46.0)	62(53.4)	0.018
Drinking	23(19.8)	57(26.5)	35(30.2)	0.237

Values are mean ± SD, n(%).

**Table 5:** The effect of monocyte count per unit increase on OS and PFS in different groups.

Risk groups	OS		PFS	
	HR (95% CI)	P	HR (95% CI)	P
GTVnx <28.5cm^3^	2.64(0.72-9.63)	0.142	2.31(1.04-5.12)	0.040
GTVnx ≥28.5cm^3^	1.94(1.25-3.00)	0.003	1.09(0.72-1.64)	0.694

## Discussion

Although the role of systemic inflammation in the development of cancer has been investigated in many studies (17), the main mechanism is still unclear. When tumors or inflammation occur in the body, monocytes in the peripheral circulation are recruited into the tumor microenvironment and further differentiate into macrophages (8) to play a functional or programmed death. Macrophages then play a role in stimulating angiogenesis, enhancing tumor cell migration and invasion, and inhibiting anti-tumor immunity in the development of tumors (18). GTV is generally considered a part of the tumor microenvironment. Still, more specifically, it represents the tumor volume visible on imaging, is a potential biological reflection of tumor burden, and is associated with the survival of tumor patients (19). Understanding the relationship between different monocyte counts and the prognosis of tumors after radiotherapy and chemotherapy under different tumor loads is conducive to the formulation of individualized treatment decisions.

In this study, it’s confirmed that pre-treatment monocyte count and GTVnx are independent predictors of survival in newly diagnosed non-metastatic nasopharyngeal carcinoma patients undergoing radiotherapy. The high monocyte count (≥ 0.29×10^9^/L) and high GTVnx (GTVnx≥ 28.5cm^3^) were associated with poor OS and PFS. At the same time, the monocyte data at 1 month, 2 months, and 3 months after radiotherapy also supported this conclusion. Moreover, correlation analysis showed that monocyte count was positively correlated with WBC, platelet, and neutrophil. GTVnx was positively correlated with platelet, neutrophil, and Epstein-Barr virus before treatment. Elevated WBC, platelets, and neutrophils are often associated with poor prognosis and may reflect systemic inflammatory responses or immune system disorders that promote tumor growth and metastasis.

After adjusting for a variety of confounding factors, for every increase in unit count of monocytes, the risk of death increases by 2.30 times. Besides, for every increase in monocyte unit count, the OS and PFS risks of patients in the low and high GTVnx groups increased by 2.64 times, 2.31 times, 1.94 times, and 1.09 times, respectively. A large number of previous studies have shown that the increase of peripheral blood monocytes is associated with poor survival of various cancers (10–13, 20–22). This is consistent with the results of our study in which NPC patients with higher baseline peripheral blood monocyte counts had worse survival outcomes than those with lower baseline peripheral blood monocyte counts. This study further verified that GTVnx and systemic inflammation, including monocytes, are important factors affecting the prognosis of NPC patients.

Monocytes can secrete various proinflammatory cytokines, which have been associated with shorter survival and worse prognosis in malignancies (9). Moreover, monocytes can also promote tumor growth and angiogenesis by releasing VEGF, epidermal growth factor (EGF), and tumor necrosis factor-α (TNF-α) (23). Peripheral blood monocyte count is closely related to tumor-associated macrophages (TAM), which differentiate into TAM through tumor microenvironment (24), and produce various cytokines and growth factors to induce tumor progression. These cytokines and growth factors can cause angiogenesis and anti-immune response. In the tumor or inflammatory site, differentiated macrophages can polarize to form different subtypes with the change of the environment, mainly divided into M1 macrophages and M2 macrophages (25). However, there is evidence that TAM in the tumor microenvironment can no longer be simply classified by the M1/M2 macrophage polarization model (26). They often have the common characteristics of both. As the tumor progresses, TAM will eventually shift to an immunosuppressive phenotype, with the characteristics of stimulating angiogenesis, inhibiting adaptive immunity, and promoting tumor growth and metastasis (27).

During radiotherapy, there is evidence that monocytes are recruited into the tumor to promote angiogenesis during radiotherapy, counteracting the therapeutic effect and ultimately negatively affecting the treatment outcome (28). When the tumor volume is large, it may also lead to changes in the tumor microenvironment, and the blood supply is difficult to reach the center of the tumor, resulting in obvious hypoxic tissue in this area, which is less responsive to radiotherapy and chemotherapy. In addition, the anatomical structure of nasopharyngeal carcinoma is complex, the wider the range of tumor invasion, the closer the distance between the tumor and the optic nerve, spinal cord, and brainstem, the greater the difficulty of radiotherapy planning, resulting in decreased efficacy and poor prognosis. A better understanding of the process of monocyte recruitment may lead to the development of new therapies to control the number and distribution of monocytes, thereby enhancing the therapeutic efficacy of malignancies and improving survival.

As one of the types of leukocyte, monocytes can be combined with GTVnx as a prognostic indicator of nasopharyngeal carcinoma due to its convenient, rapid, and low-cost detection. The results of this study showed that GTVnx ≥ 28.5cm^3^ and monocyte count ≥ 0.29×10^9^/L were adverse factors for PFS and OS. In patients with GTVnx < 28.5cm^3^, increasing monocyte count will significantly increase the risk of poor survival and disease progression. Therefore, it is imperative to develop drugs that regulate monocyte infiltration and reshape the function of TAM for clinical application. At the same time, Clinicians should be alert to the risk of local recurrence or distant metastasis and develop individualized treatment plans (such as increasing adjuvant chemotherapy, Combined immunotherapy with the targeting of tumor-infiltrating myeloid cells, etc.) for patients with nasopharyngeal carcinoma who meet these requirements according to specific conditions.

Some limitations need to be mentioned. First of all, this is a retrospective study, and inevitably there may be some bias. Furthermore, this study was conducted in one medical center, so the selection bias was inevitable. Therefore, multi-center, large-sample prospective studies are needed to further confirm our findings.

## Conclusions

In conclusion, peripheral blood monocyte count combined with gross tumor volume of nasopharyngeal carcinoma can independently predict the overall survival and progression free-survival in newly diagnosed non-metastatic NPC patients who received radiotherapy. The benefit of patients with GTVnx< 28.5cm^3^ could be remarkably attenuated by the high monocyte count. These findings should be verified in more prospective studies conducted among different populations.

## Data Availability

The raw data supporting the conclusions of this article will be made available by the authors, without undue reservation.

## Supplementary material

Supplementary Figure 1 The Kaplan–Meier survival curves of monocyte count one month **(A)**, two months **(B)** and three months **(C)** after radiotherapy based on the overall survival (OS) and progression-free survival (PFS) in NPC patients. (**A**: Monocyte LG: monocyte count <0.28×10^9^/L, Monocyte HG: monocyte count ≥0.28×10^9^/L; **B**: Monocyte LG: monocyte count <0.27×10^9^/L, Monocyte HG: monocyte count ≥0.27×10^9^/L; **C**: Monocyte LG: monocyte count <0.26×10^9^/L, Monocyte HG: monocyte count ≥0.26×10^9^/L).

Supplementary Table 1 The effect of GTVnx volume per unit increase on OS and PFS in different groups.
