# Correction: Monocyte count combined with GTVnx is an independent prognostic factor in non-metastatic nasopharyngeal carcinoma receiving radiotherapy

**DOI:** 10.3389/fonc.2025.1637309

**Published:** 2025-08-20

**Authors:** Li-Na Yang, Yun-Rui Song, Huan Zhang, Hong-Lei Tu, Ming-Yue Lu, De-Qing Liu, Jiang-Dong Sui, Dan Li, Yue Xie, Ying Wang

**Affiliations:** Radiation Oncology Center, Chongqing University Cancer Hospital, School of Medicine, Chongqing University, Chongqing, China

**Keywords:** peripheral blood monocyte count, GTVnx, nasopharyngeal carcinoma, radiotherapy, overall survival

In the published article, there was an error in the legend for **Figure 3** as published. Risk classification should be low, medium and high risk rather than no risk, low risk and high risk. The corrected legend appears below.

**Figure 3:**
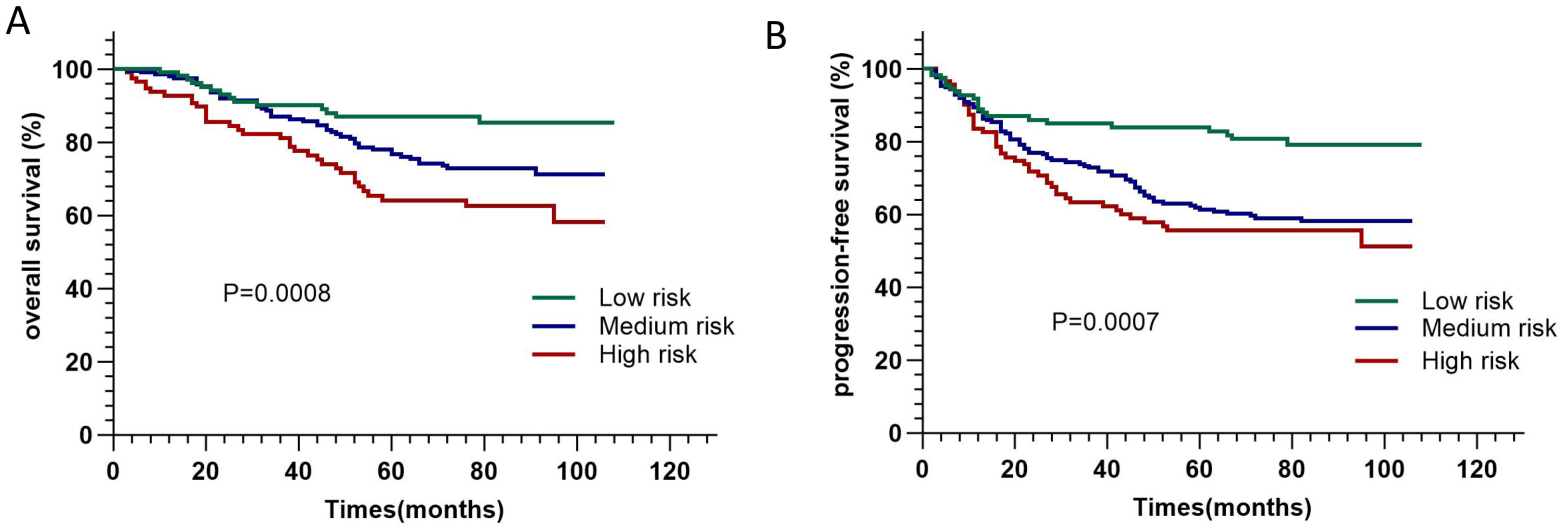
Kaplan-Meier curves of overall survival **(A)** and progression-free survival **(B)** of patients in different risk groups.

The authors apologize for this error and state that this does not change the scientific conclusions of the article in any way. The original article has been updated.

