# Supplementary material for: Monocyte count combined with GTVnx is an independent prognostic factor in non-metastatic nasopharyngeal carcinoma receiving radiotherapy
Source: Front Oncol. 2025 Apr 8;15:1541212. doi: 10.3389/fonc.2025.1541212 (PMC12011717; doi:10.3389/fonc.2025.1541212)
Supplement: Supplementary Figure 1 — The Kaplan–Meier survival curves of monocyte count one month (A), two months (B) and three months (C) after radiotherapy based on the overall survival (OS) and progression-free survival (PFS) in NPC patients. (A: Monocyte LG: monocyte count <0.28×10^9^/L, Monocyte HG: monocyte count ≥0.28×10^9^/L; B: Monocyte LG: monocyte count <0.27×10^9^/L, Monocyte HG: monocyte count ≥0.27×10^9^/L; C: Monocyte LG: monocyte count <0.26×10^9^/L, Monocyte HG: monocyte count ≥0.26×10^9^/L). [file DataSheet1.docx]

**Supplemental information for**

**High-throughput method for improving rice AGB estimation based on UAV multi-source remote sensing image feature fusion and ensemble learning**

Jinpeng Li, Jinxuan Li, Dongxue Zhao, Qiang Cao, Fenghua Yu, Yingli Cao, Shuai Feng^,*^, Tongyu Xu^**^

College of Information and Electrical Engineering, Shenyang Agricultural University, Shenyang 110866, China

National Digital Agriculture Sub-center of Innovation (Northeast Region), Shenyang 110866, China

Key Laboratory of Intelligent Agriculture in Liaoning Province, Shenyang 110866, China

**Table S1.**

Vegetation indices were selected in this study.

| Index name | Formula | Reference |
| --- | --- | --- |
| CI_red-edge_ (Red edge chlorophyll index) | (R_NIR_/R_RE_)-1 | (Cao et al., 2015) |
| GNDVI (Green normalized difference vegetation index) | (R_NIR_-R_Green_)/(R_NIR_+R_Green_) | (Rao et al., 2007) |
| MTCI (Meris terrestrial chlorophyll index) | (R_NIR_-R_RE_)/(R_RE_-R_Red_) | (Dash et al., 2004) |
| NDVI (Normalized difference vegetation index) | (R_NIR_-R_Red_)/(R_NIR_+R_Red_) | (Qi et al., 2000) |
| OSAVI (Optimized soil adjusted vegetation index) | 1.16*(R_NIR_-R_Red_)/(R_NIR_+R_Red_+0.16) | (Wu et al., 2008) |

**Table S2.**

Summary of the results of the normality test for AGB and extracted features using Shapiro-Francia at each growth stage.

| Features | P-value | | | |
| --- | --- | --- | --- | --- |
|  | Tillering | Jointing | Heading | All stages |
| AGB | 0.023885693 | 1.97E-03 | 6.59E-07 | 1.11E-11 |
| NDVI | 6.85E-07 | 3.39E-05 | 4.36E-05 | 4.11E-17 |
| MTCI | 7.70E-03 | 4.90E-06 | 7.76E-08 | 5.92E-09 |
| GNDVI | 1.72E-04 | 2.89E-06 | 6.11E-07 | 2.81E-11 |
| CIre | 3.69E-03 | 7.25E-06 | 2.01E-07 | 7.95E-09 |
| OSAVI | 0.035390999 | 2.13E-03 | 2.93E-06 | 1.29E-13 |
| haar-LH | 0.048608154 | 2.52E-03 | 0.030593812 | 0.021371253 |
| haar-HL | 0.039724551 | 0.036464464 | 3.64E-03 | 8.95E-05 |
| haar-HH | 0.033749275 | 0.038042702 | 0.014017332 | 3.20E-04 |
| db3-LH | 8.85E-05 | 3.23E-03 | 6.25E-03 | 2.61E-04 |
| db3-HL | 0.048717014 | 6.60E-03 | 0.045970866 | 0.034260079 |
| db3-HH | 1.34E-06 | 0.046788942 | 3.74E-03 | 4.06E-08 |
| sym6-LH | 0.010524774 | 0.03641839 | 5.27E-03 | 1.11E-06 |
| sym6-HL | 1.13E-06 | 0.043033265 | 0.010124288 | 1.54E-05 |
| sym6-HH | 3.15E-04 | 0.030227806 | 3.98E-03 | 1.86E-04 |
| bior3.3-LH | 8.25E-03 | 0.028586563 | 0.041021929 | 4.21E-04 |
| bior3.3-HL | 0.019092932 | 7.97E-04 | 8.68E-03 | 2.29E-03 |
| bior3.3-HH | 6.63E-06 | 0.018802213 | 8.57E-09 | 1.41E-05 |
| coif3-LH | 2.54E-03 | 0.043006006 | 0.034403287 | 8.42E-07 |
| coif3-HL | 0.032773033 | 1.74E-05 | 1.98E-05 | 1.85E-10 |
| coif3-HH | 4.85E-04 | 0.011093858 | 0.047425684 | 3.29E-05 |
| L_ave | 0.035060022 | 0.023082342 | 5.89E-03 | 0.04014441 |
| a_ave | 0.036837384 | 0.035502598 | 1.06E-03 | 6.73E-05 |
| b_ave | 0.016068723 | 1.68E-04 | 1.55E-04 | 2.28E-07 |
| L_var | 0.049039014 | 0.018100793 | 4.76E-03 | 8.29E-03 |
| a_var | 2.37E-04 | 1.97E-04 | 0.037651461 | 9.35E-06 |
| b_var | 4.85E-03 | 0.056532785 | 0.034364045 | 5.93E-03 |
| R_ave | 0.042423975 | 9.97313E-05 | 0.038872879 | 0.046699062 |
| G_ave | 0.040894784 | 0.041006044 | 0.042653706 | 4.24E-03 |
| B_ave | 0.04326015 | 0.017684789 | 0.043265294 | 3.16E-03 |
| R_var | 1.34E-03 | 4.26E-04 | 2.56E-03 | 2.45E-07 |
| G_var | 4.71E-03 | 1.55E-05 | 2.10E-03 | 4.08E-07 |
| B_var | 1.72E-03 | 4.03E-05 | 0.010285906 | 1.61E-06 |

**Table S3.**

Summary of Kruskal-Waillis test results of AGB and extracted features.

| Features | P-value |
| --- | --- |
| AGB | 4.65E-50 |
| NDVI | 6.20E-36 |
| MTCI | 2.78E-21 |
| GNDVI | 1.36E-29 |
| CIre | 2.55E-23 |
| OSAVI | 4.30E-28 |
| haar-LH | 3.62E-17 |
| haar-HL | 1.40E-16 |
| haar-HH | 1.58E-22 |
| db3-LH | 3.12E-24 |
| db3-HL | 1.28E-19 |
| db3-HH | 4.25E-26 |
| sym6-LH | 2.69E-29 |
| sym6-HL | 7.78E-25 |
| sym6-HH | 1.24E-23 |
| bior3.3-LH | 2.75E-23 |
| bior3.3-HL | 1.46E-25 |
| bior3.3-HH | 1.10E-24 |
| coif3-LH | 1.58E-19 |
| coif3-HL | 5.90E-34 |
| coif3-HH | 4.65E-23 |
| L_ave | 1.52E-18 |
| a_ave | 3.04E-37 |
| b_ave | 1.97E-08 |
| L_var | 1.02E-06 |
| a_var | 2.29E-04 |
| b_var | 2.78E-10 |
| R_ave | 5.96E-04 |
| G_ave | 3.17E-12 |
| B_ave | 9.56E-05 |
| R_var | 3.91E-09 |
| G_var | 3.79E-09 |
| B_var | 2.88E-09 |

**Table S4.**

Summary of Dunn test results of AGB and extracted features.

| P-value | Tillering-Jointing | Jointing-Heading | Tillering-Heading |
| --- | --- | --- | --- |
| AGB | 4.92E-16 | 3.78E-14 | 3.03E-51 |
| NDVI | 2.21E-30 | 0.3614913 | 5.49E-26 |
| MTCI | 1.43E-20 | 0.3017932 | 1.00E-12 |
| GNDVI | 1.44E-27 | 0.3273855 | 2.24E-18 |
| CIre | 3.38E-22 | 0.3744608 | 2.79E-14 |
| OSAVI | 1.89E-19 | 0.2008741 | 7.17E-25 |
| haar-LH | 7.69E-04 | 1.38E-07 | 6.04E-18 |
| haar-HL | 8.00E-06 | 4.40E-05 | 1.32E-17 |
| haar-HH | 2.67E-15 | 0.01678046 | 1.62E-20 |
| db3-LH | 1.00E-02 | 6.73E-16 | 2.50E-22 |
| db3-HL | 0.000005 | 0.000002 | 1.09E-20 |
| db3-HH | 4.56E-15 | 0.01151625 | 3.57E-25 |
| sym6-LH | 1.27E-18 | 5.01E-03 | 4.93E-27 |
| sym6-HL | 4.46E-19 | 6.98E-03 | 1.24E-20 |
| sym6-HH | 3.90E-04 | 4.90E-11 | 4.47E-24 |
| bior3.3-LH | 9.15E-07 | 1.28E-07 | 2.19E-24 |
| bior3.3-HL | 8.76E-07 | 8.15E-09 | 1.22E-26 |
| bior3.3-HH | 3.41E-09 | 5.01E-06 | 1.13E-25 |
| coif3-LH | 3.33E-04 | 1.69E-08 | 2.72E-20 |
| coif3-HL | 1.18E-29 | 0.01890388 | 1.59E-23 |
| coif3-HH | 4.24E-12 | 3.19E-03 | 5.21E-23 |
| L_ave | 0.8032592 | 1.63E-15 | 1.19E-14 |
| a_ave | 7.38E-10 | 2.00E-38 | 1.01E-11 |
| b_ave | 6.70E-05 | 0.06586 | 5.66E-09 |
| L_var | 2.00E-06 | 0.0627748 | 1.80E-05 |
| a_var | 0.880875 | 5.23E-04 | 2.96E-04 |
| b_var | 7.83E-03 | 4.31E-11 | 8.37E-05 |
| R_ave | 5.96E-03 | 0.335489 | 2.05E-04 |
| G_ave | 0.4079117 | 2.47E-11 | 4.99E-09 |
| B_ave | 0.010846 | 1.90E-05 | 8.39E-03 |
| R_var | 2.50E-05 | 0.063239 | 1.27E-09 |
| G_var | 1.30E-04 | 0.01916 | 6.91E-10 |
| B_var | 4.72E-04 | 5.77E-03 | 3.93E-10 |

**Table S5.**

The linear fitting results of each feature and AGB at different growth stages.

| Features | Tillering | | Jointing | |
| --- | --- | --- | --- | --- |
|  | Formulation | R^2^ | Formulation | R^2^ |
| NDVI |  | 0.43 |  | 0.60 |
| MTCI |  | 0.51 |  | 0.68 |
| GNDVI |  | 0.47 |  | 0.65 |
| CIre |  | 0.52 |  | 0.68 |
| OSAVI |  | 0.38 |  | 0.58 |
| haar-HH |  | 0.07 |  | 0.12 |
| db3-LH |  | 0.02 |  | 0.10 |
| sym6-LH |  | 0.01 |  | 0.08 |
| bior3.3-HH |  | 0.02 |  | 0.07 |
| coif3-HL |  | 0.02 |  | 0.11 |
| a_ave |  | 0.11 |  | 0.01 |
| a_var |  | 0.15 |  | 0.10 |
| G_ave |  | 0.22 |  | 0.02 |
| Features | Heading | | All stages | |
|  | Formulation | R^2^ | Formulation | R^2^ |
| NDVI |  | 0.52 |  | 0.48 |
| MTCI |  | 0.51 |  | 0.41 |
| GNDVI |  | 0.52 |  | 0.46 |
| CIre |  | 0.53 |  | 0.43 |
| OSAVI |  | 0.49 |  | 0.51 |
| haar-HH |  | 0.09 |  | 0.36 |
| db3-LH |  | 0.02 |  | 0.37 |
| sym6-LH |  | 0.07 |  | 0.39 |
| bior3.3-HH |  | 0.13 |  | 0.38 |
| coif3-HL |  | 0.05 |  | 0.35 |
| a_ave |  | 0.19 |  | 0.15 |
| a_var |  | 0.02 |  | 0.07 |
| G_ave |  | 0.04 |  | 0.13 |

**Table S6.**

The logarithmic fitting results of each feature and AGB at different growth stages.

| Features | Tillering | | Jointing | |
| --- | --- | --- | --- | --- |
|  | Formulation | R^2^ | Formulation | R^2^ |
| NDVI |  | 0.40 |  | 0.60 |
| MTCI |  | 0.51 |  | 0.68 |
| GNDVI |  | 0.44 |  | 0.65 |
| CIre |  | 0.52 |  | 0.68 |
| OSAVI |  | 0.36 |  | 0.58 |
| haar-HH |  | 0.08 |  | 0.13 |
| db3-LH |  | 0.01 |  | 0.10 |
| sym6-LH |  | 0.01 |  | 0.08 |
| bior3.3-HH |  | 0.01 |  | 0.07 |
| coif3-HL |  | 0.02 |  | 0.09 |
| a_ave |  |  |  |  |
| a_var |  | 0.16 |  | 0.09 |
| G_ave |  | 0.22 |  | 0.01 |
| Features | Heading | | All stages | |
|  | Formulation | R^2^ | Formulation | R^2^ |
| NDVI |  | 0.52 |  | 0.45 |
| MTCI |  | 0.58 |  | 0.43 |
| GNDVI |  | 0.53 |  | 0.45 |
| CIre |  | 0.61 |  | 0.45 |
| OSAVI |  | 0.49 |  | 0.47 |
| haar-HH |  | 0.09 |  | 0.37 |
| db3-LH |  | 0.04 |  | 0.36 |
| sym6-LH |  | 0.07 |  | 0.40 |
| bior3.3-HH |  | 0.12 |  | 0.37 |
| coif3-HL |  | 0.05 |  | 0.38 |
| a_ave |  |  |  |  |
| a_var |  | 0.00 |  | 0.05 |
| G_ave |  | 0.05 |  | 0.13 |

**Table S7.**

The exponential fitting results of each feature and AGB at different growth stages.

| Features | Tillering | | Jointing | |
| --- | --- | --- | --- | --- |
|  | Formulation | R^2^ | Formulation | R^2^ |
| NDVI |  | 0.46 |  | 0.64 |
| MTCI |  | 0.51 |  | 0.68 |
| GNDVI |  | 0.48 |  | 0.66 |
| CIre |  | 0.52 |  | 0.67 |
| OSAVI |  | 0.51 |  | 0.57 |
| haar-HH |  | 0.07 |  | 0.12 |
| db3-LH |  | 0.02 |  | 0.11 |
| sym6-LH |  | 0.01 |  | 0.08 |
| bior3.3-HH |  | 0.02 |  | 0.06 |
| coif3-HL |  | 0.02 |  | 0.11 |
| a_ave |  | 0.11 |  | 0.01 |
| a_var |  | 0.16 |  | 0.11 |
| G_ave |  | 0.22 |  | 0.02 |
| Features | Heading | | All stages | |
|  | Formulation | R^2^ | Formulation | R^2^ |
| NDVI |  | 0.51 |  | 0.63 |
| MTCI |  | 0.50 |  | 0.37 |
| GNDVI |  | 0.51 |  | 0.48 |
| CIre |  | 0.51 |  | 0.38 |
| OSAVI |  | 0.48 |  | 0.57 |
| haar-HH |  | 0.09 |  | 0.35 |
| db3-LH |  | 0.02 |  | 0.36 |
| sym6-LH |  | 0.08 |  | 0.38 |
| bior3.3-HH |  | 0.14 |  | 0.33 |
| coif3-HL |  | 0.05 |  | 0.31 |
| a_ave |  | 0.19 |  | 0.20 |
| a_var |  | 0.02 |  | 0.08 |
| G_ave |  | 0.03 |  | 0.14 |

**Table S8.**

The power fitting results of each feature and AGB at different growth stages.

| Features | Tillering | | Jointing | |
| --- | --- | --- | --- | --- |
|  | Formulation | R^2^ | Formulation | R^2^ |
| NDVI |  | 0.43 |  | 0.61 |
| MTCI |  | 0.51 |  | 0.68 |
| GNDVI |  | 0.46 |  | 0.66 |
| CIre |  | 0.52 |  | 0.68 |
| OSAVI |  | 0.37 |  | 0.57 |
| haar-HH |  | 0.07 |  | 0.12 |
| db3-LH |  | 0.02 |  | 0.10 |
| sym6-LH |  | 0.01 |  | 0.08 |
| bior3.3-HH |  | 0.01 |  | 0.07 |
| coif3-HL |  | 0.02 |  | 0.10 |
| a_ave |  |  |  |  |
| a_var |  | 0.16 |  | 0.09 |
| G_ave |  | 0.22 |  | 0.01 |
| Features | Heading | | All stages | |
|  | Formulation | R^2^ | Formulation | R^2^ |
| NDVI |  | 0.52 |  | 0.57 |
| MTCI |  | 0.54 |  | 0.41 |
| GNDVI |  | 0.51 |  | 0.46 |
| CIre |  | 0.57 |  | 0.44 |
| OSAVI |  | 0.48 |  | 0.56 |
| haar-HH |  | 0.09 |  | 0.38 |
| db3-LH |  | 0.04 |  | 0.38 |
| sym6-LH |  | 0.07 |  | 0.42 |
| bior3.3-HH |  | 0.12 |  | 0.39 |
| coif3-HL |  | 0.05 |  | 0.40 |
| a_ave |  |  |  |  |
| a_var |  | 0.00 |  | 0.04 |
| G_ave |  | 0.04 |  | 0.12 |

**Table S9.**

Summary of hyperparameters for the machine learning models.

| Models | Parameters | Space | Increment | Description |
| --- | --- | --- | --- | --- |
| Ada | learning_rate | 0.1-1 | 0.1 | Speed of convergence of the model gradient |
| SVR | kernel | linear/poly/RBF |  | Types of kernel functions used in the model |
|  | C | 1-10 | 1 | Penalty factors for error terms, controlling the degree of penalization for errors |
|  | tol | 0.001-0.01 | 0.001 | Residual convergence conditions for stopping training |
| GBDT | max_depth | 3-10 | 1 | Maximum depth of the decision tree |
|  | learning_rate | 0.1-1 | 0.1 | Weight reduction factor |
|  | max_features | Auto/sqrt/log2 |  | Maximum number of features considered for division |
| RF | max_depth | 3-10 | 1 | Maximum depth of the decision tree |
|  | max_features | Auto/sqrt/log2 |  | Maximum number of features considered for division |
| KNN | algorithm | Auto/ball_tree/kd_tree/brute |  | Algorithms for computing nearest neighbors |
|  | weights | Uniform/distance |  | Weighting function used in the prediction |
|  | n_neighbors | 5-15 | 1 | Number of neighbors in use |
| RR | alpha | 0.1-1 | 0.1 | Regularization strength |

Note: Ada Adaboost, SVR support vector regression, GBDT gradient boosting decision tree, RF random forest, KNN K-Nearest Neighbor, RR ridge regression

# References

Cao, Q., Miao, Y., Feng, G., Gao, X., Li, F., Liu, B., Yue, S., Cheng, S., Ustin, S.L. and Khosla, R., 2015. Active canopy sensing of winter wheat nitrogen status: An evaluation of two sensor systems. Comput Electron Agr. **112**, 54-67. <https://doi.org/10.1016/j.compag.2014.08.012>.

Dash, J. and Curran, P.J., 2004. The MERIS terrestrial chlorophyll index. Int J Remote Sens. **25**, 5403-5413. <https://doi.org/10.1080/0143116042000274015>.

Qi, J., Kerr, Y.H., Moran, M.S., Weltz, M., Huete, A.R., Sorooshian, S. and Bryant, R., 2000. Leaf Area Index Estimates Using Remotely Sensed Data and BRDF Models in a Semiarid Region. Remote Sens Environ. **73**, 18-30. <https://doi.org/10.1016/S0034-4257(99)00113-3>.

Rao, N.R., Kapoor, M., Sharma, N. and Venkateswarlu, K., 2007. Yield prediction and waterlogging assessment for tea plantation land using satellite image-based techniques. Int J Remote Sens. **28**, 1561-1576. <https://doi.org/10.1080/01431160600904980>.

Wu, C., Niu, Z., Tang, Q. and Huang, W., 2008. Estimating chlorophyll content from hyperspectral vegetation indices: Modeling and validation. Agr Forest Meteorol. **148**, 1230-1241. <https://doi.org/10.1016/j.agrformet.2008.03.005>.
